# Ants modulate stridulatory signals depending on the behavioural context

**DOI:** 10.1038/s41598-021-84925-z

**Published:** 2021-03-15

**Authors:** A. Masoni, F. Frizzi, R. Nieri, L. P. Casacci, V. Mazzoni, S. Turillazzi, G. Santini

**Affiliations:** 1 Department of Biology, University of Florence, Florence, Italy; 2 Research and Innovation Center, Sustainable Ecosystems and Bioresources, Fondazione Edmund Mach, San Michele All’Adige, Italy; 3 Department of Life Sciences and Systems Biology, Turin University, Turin, Italy; 4 Museum and Institute of Zoology, Polish Academy of Sciences, Warsaw, Poland

**Keywords:** Behavioural ecology, Animal behaviour

## Abstract

Insect societies require an effective communication system to coordinate members’ activities. Although eusocial species primarily use chemical communication to convey information to conspecifics, there is increasing evidence suggesting that vibroacoustic communication plays a significant role in the behavioural contexts of colony life. In this study, we sought to determine whether stridulation can convey information in ant societies. We tested three main hypotheses using the Mediterranean ant *Crematogaster scutellaris*: (i) stridulation informs about the emitter’caste; (ii) workers can modulate stridulation based on specific needs, such as communicating the profitability of a food resource, or (iii) behavioural contexts. We recorded the stridulations of individuals from the three castes, restrained on a substrate, and the signals emitted by foragers workers feeding on honey drops of various sizes. Signals emitted by workers and sexuates were quantitatively and qualitatively distinct as was stridulation emitted by workers on different honey drops. Comparing across the experimental setups, we demonstrated that signals emitted in different contexts (restraining vs feeding) differed in emission patterns as well as certain parameters (dominant frequency, amplitude, duration of chirp). Our findings suggest that vibrational signaling represents a flexible communication channel paralleling the well-known chemical communication system.

## Introduction

Effective communication is crucial for eusocial insects, because it allows the activity of thousands of interacting individuals to be strengthened and coordinated^1,2^. In complex societies, information is exchanged through chemical, tactile, visual and mechanical signals, the latter including both air-borne sounds and substrate-borne vibrations^3–5^. Although chemical signalling represents the primary mode of intra-colony communication in ants, vibrational communication is involved in several behavioural contexts and it is an additional method of conveying information among nestmates^6^. However, the whole spectrum of meanings of vibrational signals remains largely unclear^7–9^.

Ants can produce substrate-borne vibrations in various modalities, e.g. through whole-body movements, drumming body parts on the substrate, scraping the mandibles on the nest surface and the more specialised stridulation. Several species of the Attini tribe are hypothesized to produce alarm signals through whole body movements, termed “jigging”^10,11^, but vibrations associated with this behaviour have never been recorded^12^. Drumming behaviour, where the message is produced by tapping a body part against a substrate^7,13^ is particularly widespread within the Formicinae subfamily, especially in arboreal species belonging to the *Camponotus* genus^14–17^. The species *Dolichoderus thoracicus* and *Aphaenogaster carolinensis* were shown to produce vibroacoustic signals by scraping a substrate with their mandibles^17,18^.

**Figure 1:**
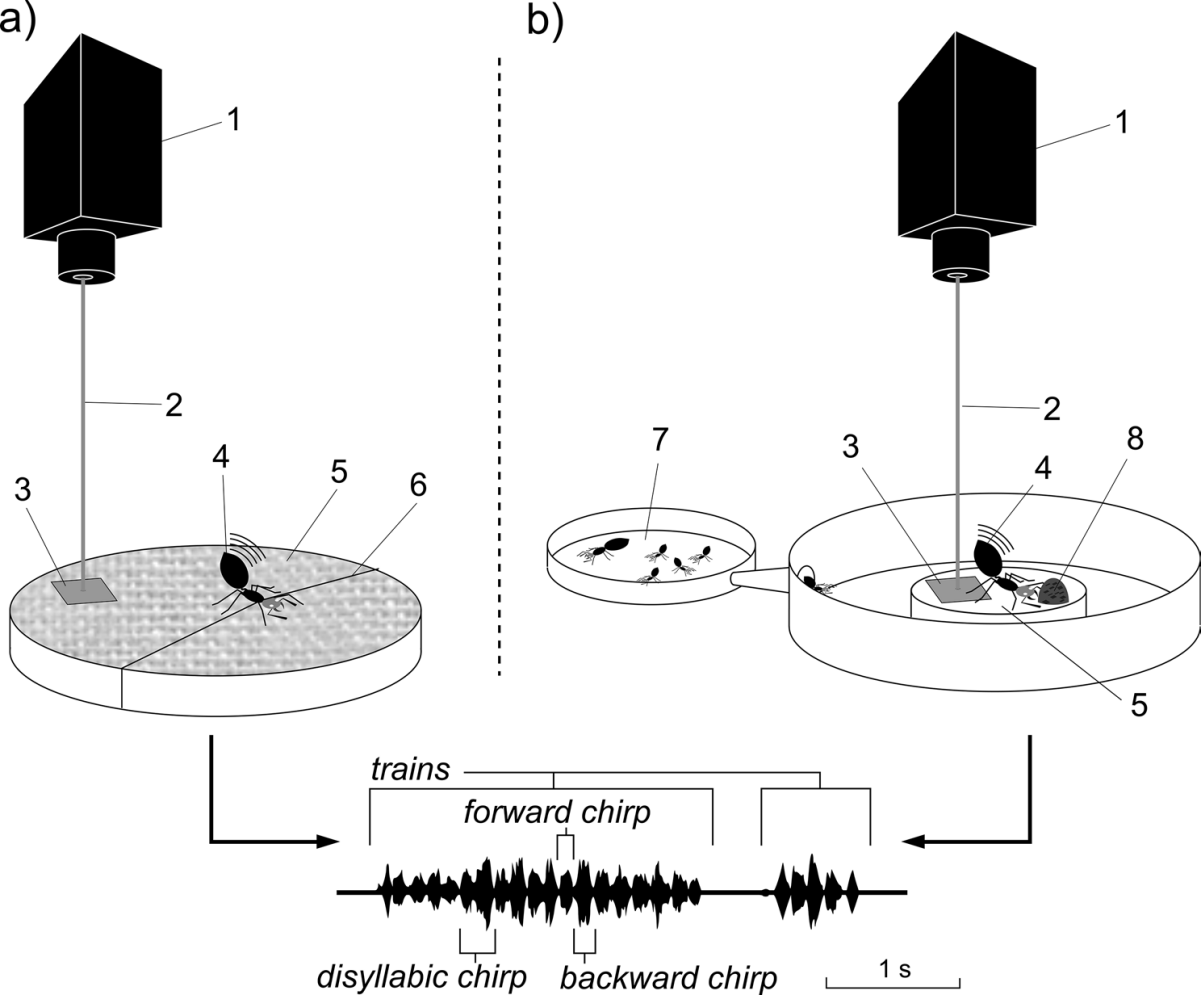
Recording set up of the restraining (**a**) and food choice (**b**) trials; 1–2 laser vibrometer, 3 reflective tape, 4 stridulating ant, 5 sheet of paper, 6 cotton thread, 7 nest arena connected to the foraging one, 8 honey drop. Below an example of an ant stridulatory signal.

Besides these modalities involving unspecialised morphological features, stridulation relies on a specialised stridulatory organ in which two sclerotised body parts are scraped together^19^. This organ has evolved multiple times in ants, and was described in almost all the studied species of the Nothomyrmecinae, Pseudomyrmicinae, Myrmicinae, Ectatomminae, Paraponerinae, and Ponerinae subfamilies^5^. It is formed by a scraper (*plectrum*), located in the upper part of the petiolar or post-petiolar tergite, and by a file (*pars stridens*), located on the upper anterior part of the first gastral segment. The stridulation is performed by rubbing the *plectrum* against the *pars stridens*, usually through the dorsoventral movement of the gaster, and the vibrations are transmitted to the substrate by the legs^20^. Sometimes ants may rotate the petiole and post petiole segments with the gaster, therefore oscillatory movement may appear on a horizontal plan. This ability clearly underline the enormous potential of the stridulatory organ to emit a large number of signals with different characteristics. A single passage of the plectrum on the *pars stridens* generates a train of pulses, which can be considered the elementary unit of stridulation and is commonly named *chirp* (Fig. 1)^2^. When the plectrum is scraped forward on the *pars stridens*, a second chirp is produced, which consists of pulses with an opposite direction with respect to the first chirp (backward chirp). The consecutive forward and backward or backward and forward scraping of the *plectrum* produces a disyllabic chirp, in which the subunits are separated by a short silence period (gap)^21^. It should be noted that both forward and backward chirp may be firstly produced from a resting position, and not all gaster movements are associated with stridulatory activity (pulse rate modulation), but ants can also move their gaster without producing vibrations, as when they spray venom or pheromones^8,22^. Therefore, stridulation is a specifically defined behavior, not associated with pheromone emission.

Vibrations and sounds, collectively called “vibroacoustic signals”, cannot be easily separated, since the same physical mechanism usually generates both at the same time. Whenever a sound/vibration source is active on a substrate, it generates both signals that transmit through the air (sounds) and signals that propagate through the substrate (vibrations)^23^. Ants are sensitive to substrate-borne vibrations, but evidence for perception of air-transmitted sounds is accumulating, at least at narrow distances^24^. Ants, like other hymenopterans and most insects, perceive substrate-borne vibrations through chordotonal organs, a type of mechanoreceptor sensilla^7,25^. Ants have two major categories of chordotonal organs, the Johnston’s organ and the subgenual organ. The first, located within the pedicel of the antennae, is sensitive to deflections of the flagellum caused by air movement, but it is not sensitive enough to perceive stridulatory sound waves^26,27^. The subgenual organ is located in the proximal portion of the tibia of all legs, and it is the primary receptor for substrate-borne vibrations in ants^28^.

It is becoming increasingly evident that stridulations may convey a considerable diversity of information among nestmates, both adult and immature ants^29^. Stridulation occurs in many different behavioural contexts, and its temporal and spectral patterns vary according to species, social organisation, and environment^30–32^. For instance, stridulatory behavior has been observed during nest excavation^33,34^, food retrieval^35^, trophallaxis and brood manipulation^20^, during conflicts^3^, as well as nest emigration^36^. Freshly mated *Pogonomirmex* queens stridulate to signal their non-receptivity to males^35^, while *Atta* workers stridulate to recruit nestmates when cutting leaves^37^. Moreover, ants stridulate when they are unable to move, as when captured by a predator or restrained by a physical impediment^38^. Nevertheless, the roles of the stridulatory behaviour in ants is not entirely clear. Only a few studies^20,39^ investigated the use of stridulation in multiple contexts within single species, and it remains to be clarified whether vibrational signals are equally used by different ant species within the same behavioural context.

According to Markl and Hölldobler^35^, vibrational signals represent one of the components of a multimodal communication system in ants, in which chemical signals directly modify the behaviour of the receiver triggering a specific response, while accessory signals, as vibrations, modulate the response. More recently, however, Barbero et al.^40^ suggested that the vibrational signals of *Myrmica schenki* can convey information on the castal status of the sender and induce specific responses in the receiving workers even in the absence of other types of stimuli. Also the fact that mutualists and social parasites imitate their host ant specific stidulations^41,42^, further supports the considerable body of evidence that recognition information are encoded in vibroacoustic signals in ants.

In this study, we aim to investigate the role of stridulation in ants focusing on the Mediterranean acrobat ant, *Crematogaster scutellaris* (Olivier 1972). This common and dominant tree-nesting ant has been chosen as a model species because the structure of the workers’ stridulatory organ is well known^43,44^, and many details of its behaviour and ecology have been recently clarified^45–49^. However, stridulatory behaviour and related substrate-borne vibrations are still unknown. In particular, we aim to answer the following questions: (1) Do the different castes emit signals that may potentially allow caste discrimination? (2) Can the stridulatory signal contain information about a specific issue, for example, the profitability of a food resource? (3) Can worker signals vary in different behavioural contexts, restraining vs. food recruitment?

We devised two sets of experiments to answer the three research questions. In the first one, we recorded the vibrations emitted during the stridulatory behaviour of either workers, males, or queens in a simulated dangerous situation, by restraining the ants. Such a condition can naturally occur, for example, during intra- or interspecific fights, predation, or when heavy debris impedes movement^50^. In the second experiment, we assessed whether stridulatory behaviour changes when foraging ants find differently sized food sources by using large and small honey drops.

## Results

Overall, the ants tested in both experiments emitted signals between 250 and 4000 Hz.

### Test 1: restraining trials

For the spectral analysis, we selected recordings with less background noise (nine queens, seven males, and nine workers). For the chirp repetition analysis, the background noise was not an impediment, thus it was possible to analyse signals from all 30 individuals recorded, ten per caste.

**Figure 2:**
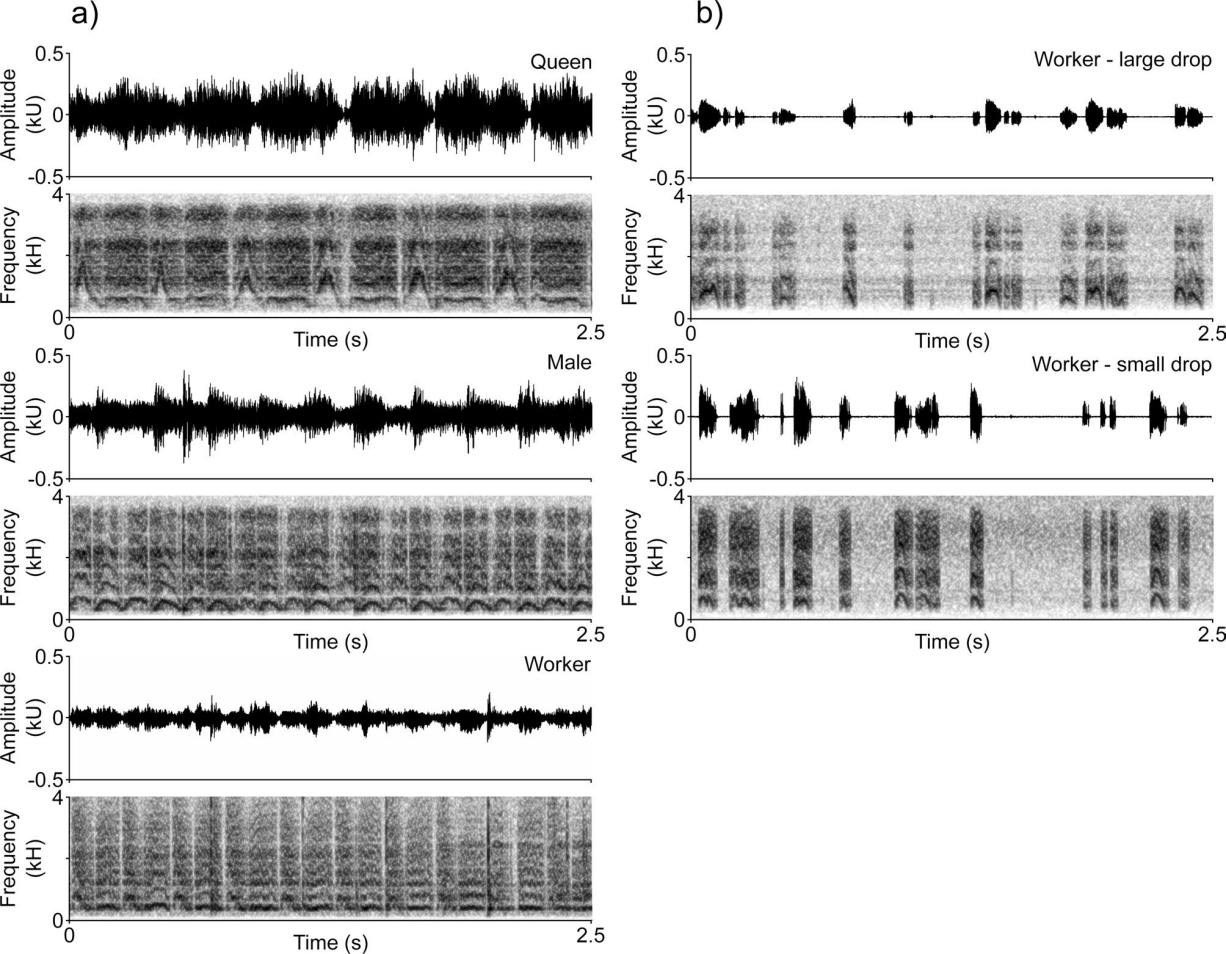
Examples of oscillograms and spectrograms of substrate-borne vibratory signals produced by *C. scutellaris* castes during restraining experiments (**a**) and by workers during food context experiments (**b**).

**Figure 3:**
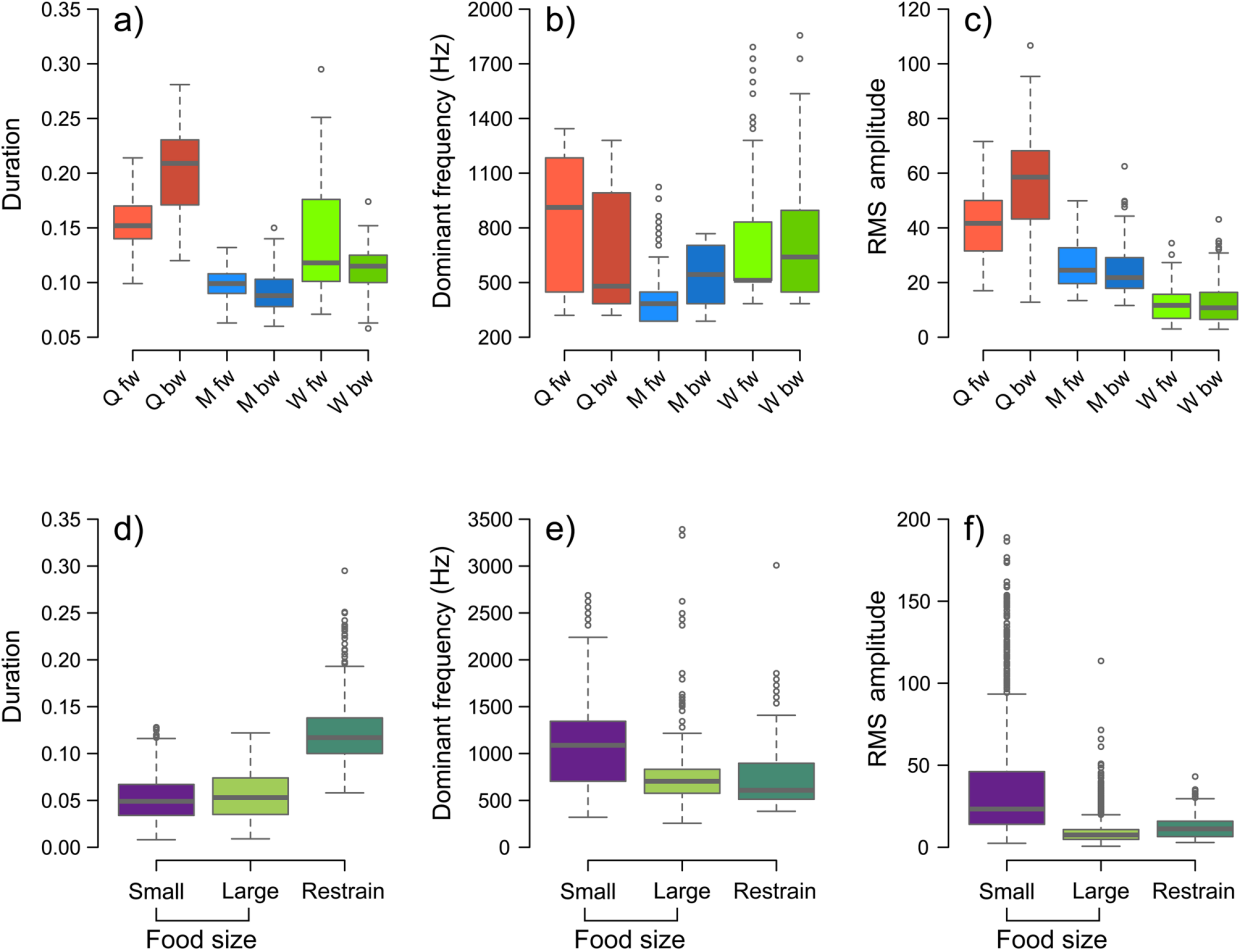
Values of dominant frequency (Hz), Root Mean Squared (RMS) and chirp duration (s) of the substrate-borne vibrations produced by stridulation, for both the restraining and food trials. Q_fw_: queens’ forward chirp; Q_bw_: queens’ backward chirp; M_fw_: males’ forward chirp; M_bw_: males’ backward chirp; W_fw_: workers’ forward chirp; W_bw_: workers’ backward chirp; Large: large honey drop (20 µl); Small: small honey drop (2 µl); Restraining: mean values of chirp parameters emitted by workers during restraining. (**a**–**c**); comparison of chirp parameter among castes; (**d**–**f**) comparison of workers’ chirp parameters between different food sizes and in the restraining context.

The overall pattern of the substrate-borne vibrations produced by the restrained ants was similar for all castes (Fig. 2a). The stridulatory emissions were characterized by variable numbers of trains of disyllabic chirps produced by the forward and backward movements of the stridulatory organ. Furthermore, in the more defined recordings each chirp unit was composed of a fundamental frequency component which in most cases also corresponded to the dominant frequency, and several harmonics. However, each caste produced distinctive signals, whose chirp units were variously modulated in frequency, intensity and duration (Fig. 2a). Indeed, the values of the dominant frequency (F_2,523_ = 83, *P* = 0.001), RMS amplitude (F_2,684_ = 492, *P* = 0.001) and chirp duration (F_1,687_ = 37, *P* = 0.001) varied significantly depending on the different castes, within the caste (Table 1) and also among different chirp units (Tables 1, 2). As to chirp duration, queens produced highly asymmetric disyllabic chirps, with forward units shorter than backwards elements, but both longer compared to males’ and workers’ syllables (Fig. 3a). Workers and queens emitted significantly longer chirps compared to males (Table 2). If we consider the frequency modulation, queens also showed the greatest variability, with the shorter unit showing a much higher frequency in the central part, compared to the backwards syllable characterised by a more constant frequency. In males chirps elements were more simmetric with visible frequency modulation, with higher frequency in the middle of the chirps. On the contrary, workers' chirps were flatter and less distinct. The dominant frequency of forward chirps (Fig. 3b) was similar for queens and workers and higher compared to males; whereas in the backward chirps, the dominant frequency was similar for queens and males, but lower compared to the workers. The RMS amplitude was higher in the sexuates, and queens emitted the most intense signals (Fig. 3c). The gap duration between forward and backward chirps varied significantly for the different castes (χ^2^_2_ = 128.8, *P* = 0.001; Table 1), but also among individuals within the castes (χ^2^_3_ = 78.8, *P* = 0.001).

**Table 1:** Mean values ± standard error of vibrational signal parameters recorded for each caste and in both behavioural contexts.

Caste	Dom. Freq. (Hz)	Sign.W	RMS amplitude	Sign.W	Chirp Duration (s)	Sign.W	Gap(s)	Sign. B
**Test 1**								
Male_F	437.62 ± 16.01	a	26.54 ± 0.71	a	0.097 ± 0.001	a	0.008 ± 0.007	a
Male_B	526.12 ± 14.64	b	24.72 ± 0.77	a	0.091 ± 0.001	a		
Queen_F	841.2 ± 40.84	a	40.87 ± 1.35	a	0.15 ± 0.003	a	0.0209 ± 0.003	c
Queen_B	644.4 ± 37.47	b	55.52 ± 2.24	b	0.203 ± 0.004	b		
Worker_F	734.43 ± 37.93	a	11.93 ± 0.56	a	0.139 ± 0.005	a	0.016 ± 0.001	b
Worker_B	758.89 ± 29.77	a	12.91 ± 0.77	a	0.113 ± 0.002	b		
**Test 2**								
Food size								
Small	1099.33 ± 26.09	a	34.10 ± 0.80	a	0.052 ± 0.001	a	–	
Large	735.68 ± 18.57	b	9.52 ± 0.24	b	0.055 ± 0.001	a		

*Sign.W* significative within caste or food size, *Sign.B* significative between castes. Common letters are not significantly different (P > 0.05).

**Table 2:** Multiple comparison among caste and between worker signals emitted in the two different contexts.

Multiple comparison between castes and tests						
Caste	Dom. Freq. (Hz)		RMS amplitude		Chirp duration (S)	
	*t ratio*	*P value*	*t ratio*	*P value*	*t ratio*	*P value*
M_fw_ – Q_fw_	− 9.04	***	− 11.52	***	− 12.47	***
M_fw_ – Q_bw_	− 4.62	***	− 22.67	***	− 23.47	***
M_fw_ – W_fw_	− 10.60	***	18.30	***	− 3.95	**
M_fw_ – W_bw_	− 11.44	***	17.56	***	2.10	N.S
M_bw_ – Q_fw_	− 7.08	***	− 12.90	***	− 13.85	***
M_bw_ – Q_bw_	− 2.73	N.S	− 24.05	***	− 24.85	***
M_bw_ – W_fw_	− 8.67	***	16.95	***	− 5.32	***
M_bw_ – W_bw_	− 9.52	***	16.21	***	0.73	N.S
Q_fw_ – W_fw_	− 1.32	N.S	23.65	***	− 12.02	***
Q_fw_ – W_bw_	− 2.04	N.S	2.06	***	11.58	***
Q_bw_ – W_fw_	− 4.81	***	32.37	***	15.42	***
Q_bw_ – W_bw_	− 5.49	***	31.78	***	20.3	***
W.R – W.SD	− 6.44	***	− 6.48	***	17.50	***
W.R – W.LD	− 0.68	N.S	6.21	***	14.46	***

*M fw and M bw* male forward and backward chirps, *Q fw and Q bw* queen forward and backward chirps, *W fw and W bw* worker forward and backward chirps, *W.R* worker restrained chirps, *W.SD* worker chirp with small food drop, *W.LD* worker chirp with large food drop, *N.S.* not significant (P > 0.05), ***P < 0.001.

**Figure 4:**
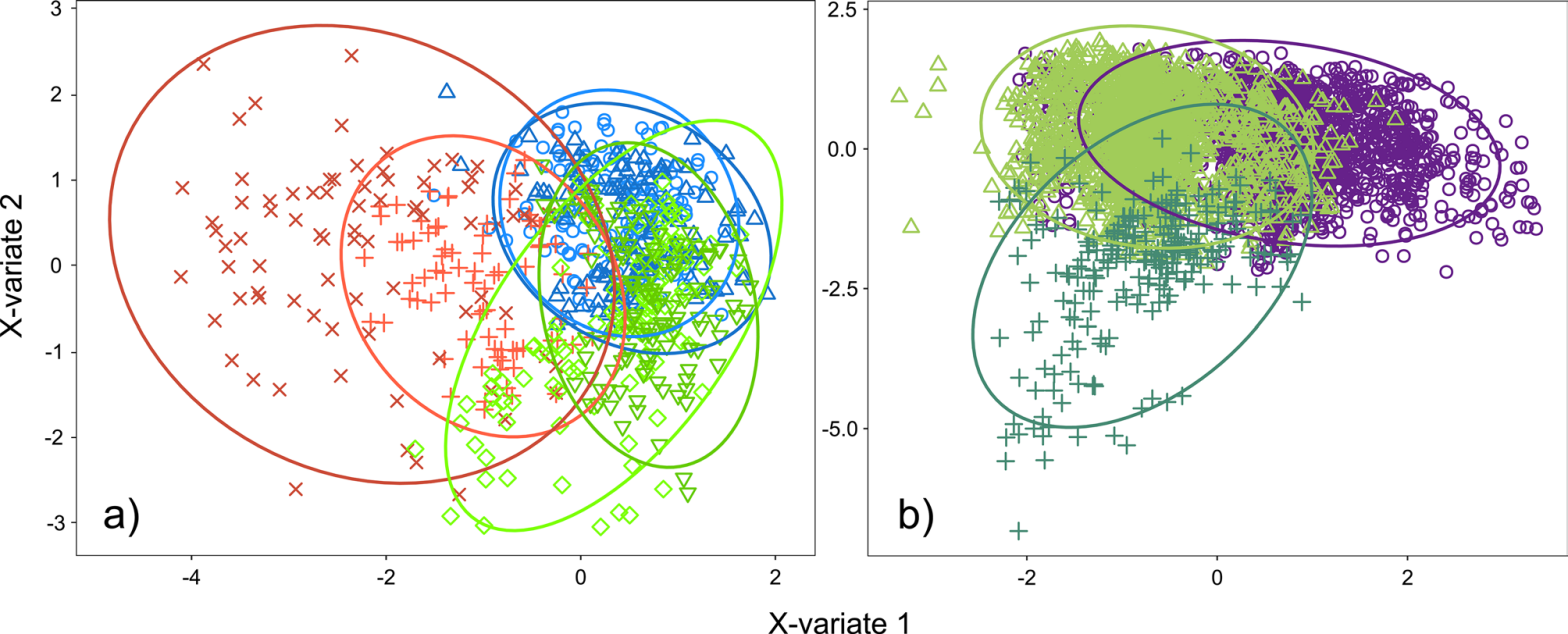
PLS-DA results for (**a**) Test 1 and (**b**) Test 2 with the comparison with workers restraining signals. Ellipses represent the 95% confidence region of occurrence of samples in one group. (**a**) plus and cross signs/red lines: forward and backward chirps of queens; circles and triangles signs/blue lines: forward and backward chirps of males; overturned triangles and diamonds signs/green lines: forward and backward chirps of workers. (**b**) Circles signs/violet line: small honey drop chirp (2 μl); triangles signs/light green line: large honey drop chirp (20 μl); plus signs/dark green: restraining chirp.

In the PLS-DA analysis, chirp duration and RMS amplitude represented the two most important parameters in both of the components (VIP values > 1 in all cases). The chirp units largely overlapped among the castes (Fig. 4a) and especially between males and workers, while the queens’ chirp units remained partly isolated. The npMANOVA showed a significant effect of the factors ‘caste’ (F_2,852_ = 290.71, *P* = 0.001) and ‘chirp type’ (F_3,115_ = 26.33, *P* = 0.001), although there was significant interindividual variation (F_6,110_ = 12.51, *P* = 0.001).

**Figure 5:**
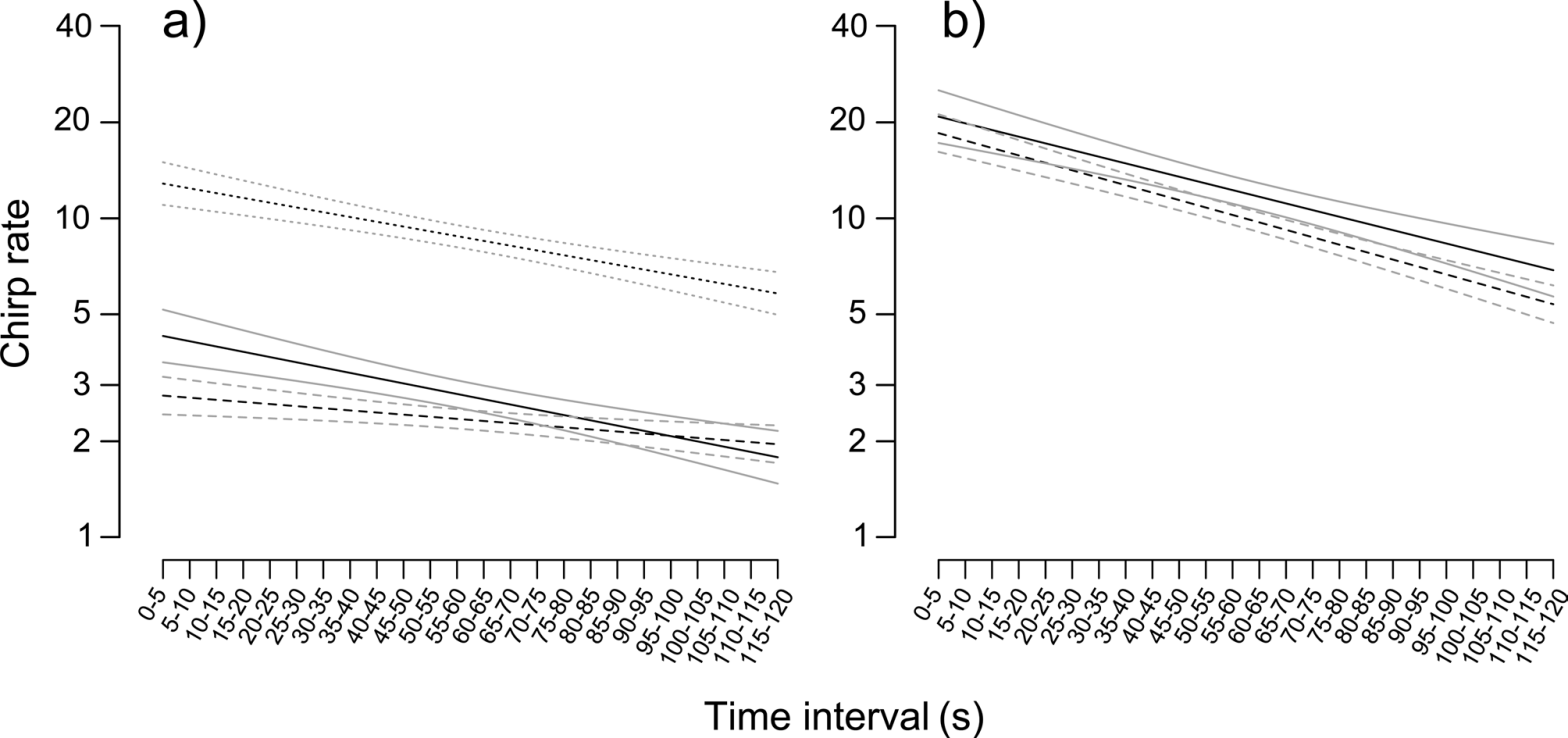
Temporal dependence of the number of chirps produced for each of the 24 five-second consecutive intervals (*chirp repetition*) for both Test 1 (**a**) and Test 2 (**b**), with their models fitted (black lines) and the standard errors of predicted values (grey lines). Test 1 (**a**): dashed lines, queens; solid lines, males; dotted lines, workers. Test 2 (**b**): solid lines, small honey drops; dashed lines, large honey drops.

In general, chirp repetition significantly decreased over time (Table 3), with queens showing the lowest decreasing trend of the model compared to males and workers (Fig. 5a). Moreover, chirp repetition was significantly higher in workers than in the other two castes, in which no differences were found.

**Table 3:** Results of mixed models for the chirp repetition, both for Test 1 and Test 2.

Test 1 (A)	df	DenDF	F value	*P*
Time	1	689	34.705	< 0.001***
Caste	2	27	5.426	0.0104*

Test 1 (B)	Q vs M	Q vs W	M vs W	
z	− 0.389	− 3.027	2.638	
*P*	0.92	0.007**	0.023*	

Test 2	Df	DenDF	F value	*P*
Time	1	920.98	826.583	< 0.001***
Food size	1	35.98	0.9146	0.345

Test 1-A and Test 2: type III ANOVA with Satterthwite’s method, Test 1-B: multiple comparisons between castes in pair (Tukey contrasts). Asterisks indicate the level of significance (*< 0.05; **< 0.01). *Q* queens, *M* males, *W* workers, df and DenDF are the two freedom degrees of the model.

### Test 2: food trials

Workers started to stridulate as soon as they began to consume the honey. All the recorded signals consisted of a series of separated chirps, not clustered in trains. Chirps appeared discontinuous, of variable duration and consisting with harmonic structure. In addition, they were characterized by a strong frequency modulation which gave the signals a characteristic inverted "U" or “M” appearance. Examples of chirps recorded from ants feeding on small and large honey drops are shown in Fig. 2b.

The values of the dominant frequency and RMS amplitude (Table 1) varied depending on the honey drop size, whereas chirp duration (Fig. 3d) did not significantly differ (F_1,38_ = 0.13, *P* = 0.720). Workers produced signals with a higher dominant frequency (F_1,31_ = 22.5, *P* = 0.001) and RMS amplitude (F_1,31_ = 23.5, *P* = 0.001) when they were fed with the small honey drop (Fig. 3e–f).

In the PLS-DA analysis, the signals produced by workers feeding on small (2 µl) or large (20 µl) honey drops were clearly distinguishable (Fig. 4b), and this difference is significant (npMANOVA F_1,16_ = 6.30, P = 0.001). In addition, these differences are explained by the dominant frequency and RMS amplitude values of the signals, as it was explained by first two components (VIP values ≥ 1 in both components).

As with the restraining trials, the chirp repetition emitted by the ants significantly decreased over time, whereas no difference was found to depend on food size (Fig. 5b; Table 3).

### Comparison between Test 1 and Test 2

Workers produced structurally different signals in the two behavioural contexts. In the restraining context, the recorded signals were characterized by trains of disyllabic chirps separated by gaps of silence (Fig. 2a). In the presence of honey drops, the stridulatory emissions were instead made up of successive single chirps of variable duration not clustered in trains and spaced by variable intervals of silence (Fig. 2b). The dominant frequency, RMS amplitude, and chirp duration significantly varied depending on the context (F_2,1251_ = 175.3, *P* = 0.001; F_2,1833_ = 848.2, *P* = 0.001; F_2,753_ = 175.9 *P* = 0.001; respectively for restraining vs. food comparison) (Fig. 3d–f). The signals produced while feeding on small honey drops showed a significantly higher dominant frequency (Fig. 3e) than that of the signals emitted while feeding on large honey drops and being restrained (Table 2). Chirp duration and the RMS amplitude of the signals significantly varied between all analysed contexts (Fig. 3d–f, Table 2). In the PLS-DA analysis, the signals produced by workers feeding on small and large honey drops, and during restraining appeared to be clearly distinguished (Fig. 4b), and this difference was significant (npMANOVA F_1,16_ = 6.30, *P* = 0.001).These differences are explained by the dominant frequency and RMS amplitude values in the first component (VIP values ≥ 1 ) and chirp duration and RMS amplitude in the second component (VIP values ≥ 1).

## Discussion

The results of this study contribute to shedding light on the role of stridulatory communication in ants and provide robust evidence that the stridulations can be regarded as real vibroacoustical signals emitted by individuals to communicate relevant pieces of information to nearby conspecifics. In particular, the key findings can be summarised as follows. First, queens, males, and workers of *Crematogaster scutellaris* actively stridulate and, in particular, queens produce vibrations qualitatively different from those of the other castes (question #1). Second, the properties of the vibrations emitted by workers during foraging activity change depending on the size of the food source they were exploiting (questions #2). Third, when engaged in different activities, workers produce different type of vibrations (question #3).

All the recorded vibrations had most of their energy in the 250–4000 Hz frequency range, which corresponds to the sensitivity of the subgenual organ in ants^51^. Differences among the castes are similar to those described for the four *Messor* species^52^, *Myrmica schenki* and *M. scabrinodis*^40,53^. The queen’s vibratory signals, which are distinct from worker and male emissions, could contain information on the rank of the emitter, as this also happens in *Myrmica* ants^53^. The marked differences in several of the properties of the vibratory signals among queens, males and workers may indicate the caste of the emitting individual during threatening situations and, for example, determine rescue priorities after nest perturbations. However, this scenario may be even more complex, and sex-related differences in stidulatory signals could be important during mate selection, copulation or postcopulatory behavior at the mating leks or in preparation for nuptial flight^35^. The observed differences can reasonably be explained by the morphological differences of their stridulatory organs^43,44^ (see Supplementary Materials, organ morphology section). Indeed, the organs of both queens and males differ from those of the workers, being generally larger and elliptical in the sexuates, and smaller and less elongated in the workers (Fig. S2). The dominant frequency of the signal is expected to vary with the width and spacing of the ridges, chirp duration mostly depends on the length of the *pars stridens*, while chirp repetition reflects the speed and rhythm of scraping the plectrum on the file^40^. Additionally, the duration of individual chirps may vary if the ant rubs the scraper only on a portion of the pars stridens^53,54^.

A key finding of this study is that workers are able to produce stridulations that vary with the amount of food encountered. *C. scutellaris* workers seem able to discriminate the size of the honey drop and to respond by generating different kinds of signals. The ability of ants to discriminate food size and change their behaviour accordingly is not surprising and is known from other ant species. For example, leaf-cutting ant *Acromyrmex lundi* foragers cut different sizes of leaf fragments depending on the amount of food they find^55^, and polymorphic harvester ants optimize foraging efficiency by selecting seeds by their shape and size^56^. The termite *Cryptotermes secundus* directly uses vibrational signals to evaluate food size, i.e. the dimensions of the trunk, triggering adequate behavioural responses from colony members^57^. It is possible that *C. scutellaris*, whose workers individually search for food, recruit conspecifics even several meters away from the nest depending on its concentration^58^, which may involve stridulatory signals in parallel to chemical communication, as has been suggested to occur in *Leptothorax muscorum*^20^. Nevertheless, the effectiveness of stridulatory signals in nestmate recruitment needs to be assessed in more detail with playback experiments aimed at analysing the behavioural response of the receivers. Playback experiments can also evaluate the distance at which the signals are effective. A key point worth investigating is the propagation and attenuation of vibrations in different substrates, as their physical properties can considerably affect signal transmission, and hence effectiveness^59,60^. The transmission of vibrational signals by plants has been extensively investigated, where they are mainly transmitted as boundary waves that are considered to be dispersive^50,61^. However, a recent study showed that depending on the size of the plant stem, bending waves transmit as dispersive waves for low frequencies and as non-dispersive waves for higher frequencies^62^. The transmission of vibrational signals in soil is even more complex because it must take into account the dimension of the grains and the content of water and air^19,61^. Considering the different allocation of energy in signals emitted by workers feeding on small honey drops (dominant frequency: 1099 Hz) compared to large ones (dominant frequency: 735 Hz), we hypothesize that the two signals may be transmitted differently depending on the distance from the source. However, more tests aimed at identifying what kind of waves are involved in *C. scutellaris* communication, their propagation across the substrate, and the active space of the signals must be conducted to verify that the difference in the dominant frequency of the two signals determines a different range of transmission in the substrate. At the moment, we cannot exclude that in *C. scutellaris* a component of the signal is transmitted thorugh air, as it has been shown in other ant species in which stridulations were recorded holding individual ants with tweezers at a distance of 1 cm from a microphone^20,30^. Such component would add an additional level of complexity to the signal that is worth testing in future studies.

In both tests, chirp repetition rapidly decreased over time. In a dangerous situation, such as that represented by the restraining trials, it is possible that the ant, immediately after entrapment, responds with a ‘panicky’ reaction, usually associated with an upsurge in activity^63,64^. Later on, the ant probably reduces its movement to save energy. In the food trials, such behaviour could instead be due to the excitement of the forager when it finds a new resource^8,65^, which quickly decreases during feeding activity.

Finally, it is important to note that workers stridulate differently depending on the specific context they are experiencing. The signals generated during the restraining trials were structurally very different from those emitted during the feeding test. In the former, the signals generally consisted of sequences of dysillabic chirps usually organised in trains, whereas in the latter, the signals consisted mostly of isolated monosyllabic chirps of variable duration and without any evident temporal regular pattern. Although there are evident differences in the patterns of signal emission and the type of signal units (disyllabic chirps and monosyllabic chirps in the presence of food) depending on the context, the chirp repetition of workers appeared to be very similar in the two contexts. This finding contrasts with the behaviour of the ant *Leptogenys kitteli*, whose workers produced signals at higher rates in the presence of prey or when disturbed with respect to the signals emitted during routine activities, such as social interactions and feeding within the nest^31^. At any rate, another important aspect about the variability of *C. scutellaris* stridulation concerns the ability of single individuals to modulate signal parameters within the same context. The duration of the signals could be modulated by slowing down or accelerating the movement of the abdomen which produces the rubbing of the pars stridens on the plectrum. Instead, the frequency modulation and the distinct structure of signals could result from the possibility of rubbing the ridges of the pars stridens, not only by moving the organ up and down, but also to the right and left, thus altering the pulse emission over time. It is therefore evident that the stridulatory organ represents a highly versatile tool, which can be used by any colony individual to potentially produce a variety of different signals. In this way, stridulatory signals may convey information about the context in greater depth. In a communicative perspective, restraining signals should alert nestmates about possible dangers or could represent a call for help, similarly to the substrate-borne vibrations described for termites^66^, or the drumming behaviour of *Camponotus* ants^2^. Food signals should recruit workers that may be foraging close to the food source^37^ and potentially inform them about the amount of food.

The use of vibroacoustic signals has long been considered a limited form of communication, exclusive to certain contexts and uses, such as threatening situations or for the modulation of chemical signals^1,17,57^. However, it is increasingly evident that, although possesssing a rather simple instrument—the stridulatory organ, ants are able to modify the pattern of vibroacoustic signals, modulating temporal characteristics, such as the duration and repetition rate of the signals, but also the characteristics of frequency and intensity. These differences do not only concern individuals belonging to different castes, as already demonstrated in other studies, but it is clear that each individual within a colony can produce signals with diverse characteristics depending on the behavioural context, conveying a high and as yet little known amount of information to colony nestmates. In conclusion, it is likely that vibrational communication represents a more flexible channel of ants’ communicative systems, as relevant as chemical communication.

## Materials and methods

### Recording equipment

To date, it has been shown that ants appear to be insensitive to the aerial component^27^ but highly responsive to the vibrational component of signals^67,68^. Therefore, all our recordings were made considering only the substrate-borne signals.

All recordings were made on an anti-vibration table in a soundproof room at a constant temperature (25 °C) and relative air humidity (70 ± 5%) at the Laboratory of Biotremology of Fondazione Edmund Mach (Trentino, Northern Italy). Stridulatory behaviour and substrate-borne vibrations were simultaneously recorded using a laser vibrometer (Ometron VQ-500-D-V, Harpenden, UK) connected to a camcorder (Panasonic HDCTM700, Hamburg, Germany) as an external microphone. The camcorder was equipped with a macro lens (Raynox dcr-25) (See Supplementary Materials for a sample video, Video S1). A 4 × 4 mm reflective tape was attached to the center of the recording arena and used to reflect the focused beam of the laser vibrometer (Fig. 2). An approximate distance of 1 cm between the stridulating ant and the reflective tape was maintained as fixed for all tests. During the recording, vibrations were digitized with a sampling rate of 16 kHz and 16-bit resolution and stored on a laptop computer using LAN-XI data acquisition hardware and Pulse 18 software (Brüel and Kjær Sound and Vibration A/S, Nærum, Denmark). The bandwidth of the recordings was 8 kHz.

### Test 1: restraining trials

Ten ants of each caste (queens, males, and workers) were recorded. Workers were collected from different colonies, whereas freshly mated, dealate queens and winged males were collected during nuptial flights in the early days of September in San Michele all’Adige, northen Italy (46.193992N, 11.135045E). Each ant was maintained in isolation in a Petri dish (2 cm diameter) with a moistened cotton ball to provide sufficient humidity. During the same days, ten groups of workers (ten individuals per group) were collected from 10 different colonies and housed in 9 cm diameter Petri dishes providing the same humidity conditions. All the specimens were maintained in a thermostatic chamber in dark conditions and a constant temperature (25 °C) for 24 h before being used in the experiment. The experimental arena consisted of a sheet of white paper (80 g/m^2^ grammage) stretched and glued on the top of a 9 cm diameter plastic ring (Fig. 1a). The paper was replaced after each test to prevent any influence from the chemical traces left by the ants. One individual ant was restrained in the middle of the arena by blocking its head with a cotton thread that allowed the ant to move its gaster and thus freely stridulate. Ants started to stridulate as soon as they were restrained; their behaviour and associated vibrations were recorded for two consecutive minutes with the laser vibrometer and the camcorder (see the “Recording equipment” section).

### Test 2: food trials

Workers were randomly collected from ten 2-years-old *C. scutellaris* colonies that were reared in the laboratory at the University of Firenze science campus in Sesto Fiorentino (Florence, Central Italy, 43.817214 N, 11.204264 E) since the queen founding phase. Each colony was formed by a single queen, 40–45 workers of normal dimensions (average cephalic width 1.01 mm, see Supplementary Material, Table S1), few nanitic workers, smaller in size (average cephalic width 0.74 mm, the first workers to be born), and all brood stages. The colonies were housed within darkened 4 cm diameter Petri dishes connected to a foraging arena (15 cm diameter) with a narrow plastic tube (Fig. 1.b). The walls of the foraging arena were coated with Fluon (fluoropolymer resin, PTFE-30) to prevent the ants from escaping. All colonies were fed with honey and *Drosophila* adults, but they were maintained without food for ten days before being used in the experiment.

During the trial, honey was used as food source, and small (2 µl) or large (20 µl) droplets of honey were provided. At the beginning of the experiment, one drop, either small or large, was laid in the middle of the recording arena, made as previously described in test 1, but smaller in size (4 cm diameter). The Petri dish with the ants was then placed inside the foraging arena, and as soon as the first adult workers discovered it (usually within 1 min), it was carefully moved on the recording table. As soon as the dish was placed on the table, the recording of vibrations started and lasted for at least 2 min. This experimental procedure was set up in order to record only those ants that spontaneously moved out from the nest in search for food (foraging workers), without stressing them by forceps manipulation and avoiding multiple ant interaction at the food source. We recorded 20 foraging ants for each droplet size, for a total of 40 workers from the reared colonies (n = 10).

Each day we tested an equal number of small and large honey drops, using ants from different colonies. The recording arena was replaced at the end of each test.

### Signal analysis

The vibroacoustic analysis of stridulations recorded with the laser vibrometer as audio files was performed with Raven v1.5 software (Bioacoustics Research Program, Cornell University, Ithaca (NY), 2014). From the recordings produced during the restrained assays, we discarded the parts of the signals evidently blurred by the noise produced by the ant’s body movements while attempting to free itself and applied a high-pass filter, with a cut-off frequency 120 Hz, to reduce the low-frequency noise. Filtered signals were analyzed with a Fast Fourier Transform (FFT) (type Hann, window length of 256 points and 75% of overlap). A variable number of trains (from one to 16) per individual were selected and a variable number of disyllabic chirps (from one to 14) per train were analysed. In the recordings obtained during the food trials, all chirps emitted during the first 15 s were selected for analysis. An example of a stridulatory signal is presented in Fig. 1 together with the two types of chirps that were identified depending on the gaster movement generating them (forward, chirp “F”; backward, chirp ”B”). To describe and compare the structure of the vibrational signals, we measured the following parameters for each unit of the disyllabic chirps emitted during the restraining trials and for single chirps produced during the food trials: (i) *dominant frequency* (Hz): the frequency at which the maximum power occurs within the average spectrum of a chirp; (ii) *RMS amplitude*: the Root-Mean-Squared amplitude (RMS) of the signal (sometimes called “effective amplitude”), this represents an index of the amplitude of a signal and is dimensionles^69^; (iii) *chirp duration* (s): time interval from the onset to the end of a chirp.

For each disyllabic chirp produced during the restraining trials, we additionally measured the (iv) *gap duration*(*s*) (duration of the interval between two syllables of the opposite phase).

For each 2-min recording obtained from the restrained ants and during the food trails, we also calculated (v) the *chirp repetition* as the number of chirps counted for each of the 24 five-second consecutive intervals.

### Statistical analysis

Data were analysed using a combination of univariate and multivariate analyses. Because of the large difference in scale, variables were log_10_ (+ 1) transformed, and then normalized to zero mean and unit variance before performing the analyses.

All variables, except the chirp repetition, were separately analysed using Generalised Linear Mixed Models (GLMMs). For signals emitted during Test 1, models for variables *i, ii,* and *iii* included the caste, chirp type (forward; backward) and their interaction as the main factors, and the individual as the random factor. Since interaction was found to be significant (see results), we performed multiple comparisons between the interaction levels, i.e. each combination of the two main variables is a level of the new factor. We calculated the Estimated Marginal Means (EMMs) for all these levels, and we used them for multiple comparisons between the levels in pairs. For *iv*, the model included the caste as the main factor and the individual as the random factor. Multiple comparisons between the castes were then performed as above. For signals produced during Test 2, models for variables *i*, *ii*, and *iii* included food size as the main factor and the individual as the random factor. Here, *iv* was not included in the analysis, since the emitted signals were not organised into disyllabic chirps (see results).

Univariate analysis was also used to compare the parameters of the signals emitted by workers during restraining and food trials. A Generalised Linear Model (GLM) considering the behavioural context of the signal (restraining or food) was fitted for only three variables (*i-iii*). The *gap duration* was not included for the reason described above.

The multivariate analysis was applied to assess the differences in the vibroacoustic chirps using the first three parameters (*i–iii*) as response variables, either for signals produced during Test 1 and Test 2, and to compare worker signals emitted in the two contexts. First, we performed a Partial Least Square Discriminant Analysis (PLS-DA)^70^, using the Euclidean distance as a measure of dissimilarity. Then, the significance of the differences among the groups (i.e. chirp type and caste in Test 1, food size in Test 2, and the comparison of signal types among workers) was assessed using a permutation-based nonparametric multivariate analysis of variance (npMANOVA), as described in Anderson^71^.

Finally, to analyse the temporal distribution of chirp repetition (*v*) we log_10_ (+ 1) transformed the *chirp repetition* of each bin and used a Linear Mixed Model (LMM) both for Test 1 and Test 2, where time is the main factor and the individual the random factor. For all the statistical analyses, we used R v. 3.6.1 software^72^ with the “mixOmics”^73^, “vegan”^74^, “BiodiversityR”^75^, “multcomp”^76^ and “lmerTest”^77^ packages.

## Supplementary Information

Supplementary Information.

Supplementary Video S1.
