# Supplementary material for: Ants modulate stridulatory signals depending on the behavioural context
Source: Sci Rep. 2021 Mar 15;11:5933. doi: 10.1038/s41598-021-84925-z (PMC7970987; doi:10.1038/s41598-021-84925-z)
Supplement: Supplementary file 1 — Supplementary Information. [file 41598_2021_84925_MOESM1_ESM.pdf]

Ants modulate stridulatory signals depending on the behavioural context

Masoni A.[1*†], Frizzi F.[1†], Nieri R. [1,2], Casacci L.P. [3,4], Mazzoni V. [2], Turillazzi S. [1], Santini G. [1]

[1]Department of Biology, University of Florence, Florence, Italy
[2]Fondazione Edmund Mach, Research and Innovation Center, Sustainable ecosystems and bioresources, San Michele all'Adige, Italy
[3] Department of Life Sciences and Systems Biology, Turin University, Turin, Italy
[4] Museum and Institute of Zoology, Polish Academy of Sciences, Warsaw, Poland
[*]Corresponing author: alberto.masoni@unifi.it
[†] Equal contribution

SUPPLEMENTARY

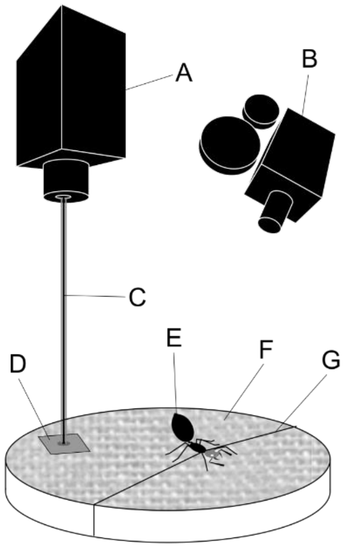

Figure S1. Experimental apparatus for the restraining trial. A) laser vibrometer; B) camera recorder; C) laser beam; D) reflecting surface; E) ant; F) arena surface (paper); G) cotton thread.

Morphology of the stridulatory organ

A comparative description of the stridulatory organ was made based on SEM photographs from the three different castes. Two newly mated queens, two winged males, and two adult workers dry mounted or preserved in 70% alcohol, were cleaned with pure ethanol and dissected under a stereomicroscope to expose the *pars stridens* and the *plectrum*. The samples were mounted on aluminium supports (stubs) covered by conductive glue, and sputter-coated with graphite and gold. Prepared samples were observed using a Zeiss Evo MA15 Scanning Electron Microscope (SEM) at the Interdepartmental Center for Electron Microscopy and Microanalysis (M.E.M.A.) of the University of Florence. All measurements of the different parts were taken from SEM photographs using the software package Image J ver. 1.46r [69]. We measured the following morphometric variable: head width (measured above the eyes, mm), width and length of the pars stridens (μm) and width of the ridges of *pars stridens*. The width of the ridges was obtained as the average of the size of ten ridges for each ant. Given the small sample size, no statistical tests were performed on these data.

Examples of *pars stridens* for each caste are shown in Figure S.2. Table S1 shows the measurements (average and standard deviation for all castes) of the considered morphometric characters. The *Plectrum* is formed by a thickening of the back edge on the third abdominal tergite

and does not involve any other modified structure (Fig.S3). The *pars stridens* is an elliptical or oval area, which occupies the medial region of the fourth abdominal pretergite, and characterised by thin parallel ribs, conferring a uniformly striated aspect (Fig.S2). The stridulatory organ of workers presented a pars stridens with a regular oval shape (Fig.S2a), with similar length and width. Queens (Fig.S2b) have a pars stridens with an elliptical shape, being much longer than wide, with an average ridge width of 0.92µm. The stridulatory organ of males is similar in shape to that of the queens, although smaller in size and with thinner ridges (Fig. S2c).

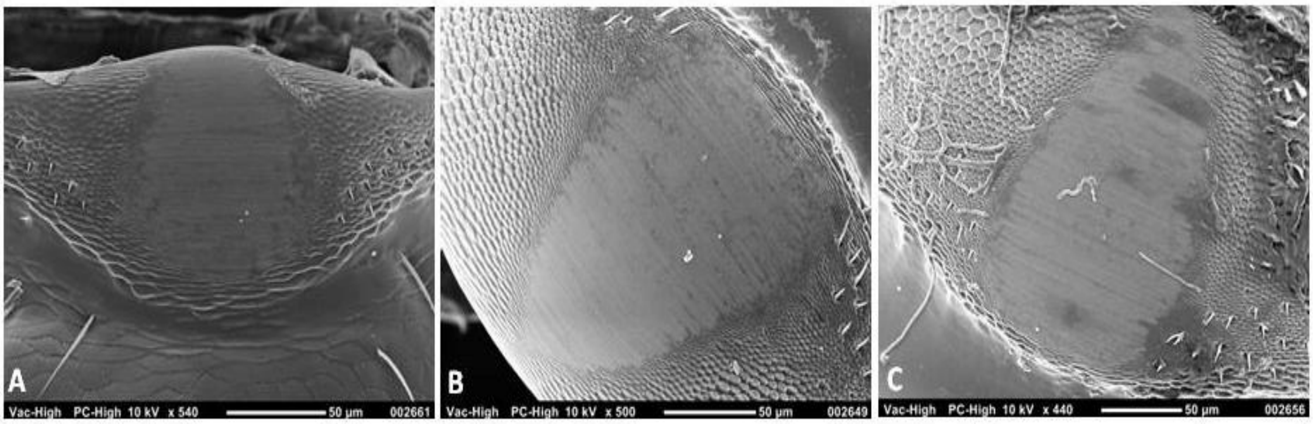

Fig. S2 Scanning electron microscopy photograph of the pars stridens of different *C. scutellaris* castes. (A) worker, (B) queen, (C) male.

		Head width (mm)		Lenght (µm)		Width (µm)		Ridge width (µm)	
**Caste**	n	Average	SD	Average	SD	Average	SD	Average	SD
Worker	2	1.03	0.05	101	4.1	93.3	9.1	0.73	0.05
Queen	2	1.54	0.03	170	11.5	128	6.2	0.92	0.09
Male	2	0.64	0.06	153	5.2	132.5	4.2	0.83	0.07

Tab.S1 Average and standard deviation (SD) of the morphometric variables of the three castes.

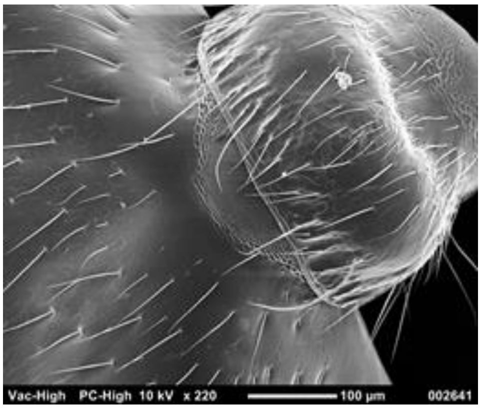

Fig.S3 Scanning electron microscopy photograph of a queen's *plectrum* connected with the *pars strindens*.

References

69. Schneider, C. A., Rasband, W. S., & Eliceiri, K. W. 2012. NIH Image to Image J: 25 years of image analysis. *Naturalistic methods*, 9(7), 671-675.
